# The After-Effect of Accelerated Intermittent Theta Burst Stimulation at Different Session Intervals

**DOI:** 10.3389/fnins.2020.00576

**Published:** 2020-06-25

**Authors:** Fengyun Yu, Xinwei Tang, Ruiping Hu, Sijie Liang, Weining Wang, Shan Tian, Yi Wu, Ti-Fei Yuan, Yulian Zhu

**Affiliations:** ^1^Department of Rehabilitation Medicine, Huashan Hospital, Fudan University, Shanghai, China; ^2^School of Kinesiology, Shanghai University of Sport, Shanghai, China; ^3^Shanghai Key Laboratory of Psychotic Disorders, Shanghai Mental Health Center, Shanghai Jiao Tong University School of Medicine, Shanghai, China; ^4^Co-innovation Center of Neuroregeneration, Nantong University, Nantong, China

**Keywords:** theta burst stimulation, accelerated, motor cortex, cortical plasticity, stimulation interval

## Abstract

**Objective:**

The study aims to investigate the after-effect of three sessions of intermittent theta-burst stimulation (iTBS) on motor cortical excitability. The iTBS was induced over the primary motor cortex (M1) at different time intervals.

**Methods:**

The study has a crossover design. Sixteen participants were assigned to three groups and received different accelerated iTBS (aiTBS) protocols during each visit: (1) three continuous sessions with no interval (iTBS18000); (2) three iTBS sessions with 10-min intervals (iTBS600 × 3^∗^10); and (3) three iTBS sessions with 30-min intervals (iTBS600 × 3^∗^30). As washout period, each visit is separated by at least 7 days. We measured the motor cortical excitability changes and intracortical inhibition.

**Results:**

A dose of 1,800 pulses of aiTBS per day is tolerable. The iTBS1800 led to a reduced cortical excitability; whereas iTBS600 × 3^∗^10 and iTBS600 × 3^∗^30 enhanced cortical excitability to a differential extent. After a total dose of 1,800 pulses, iTBS600 × 3^∗^30 exhibited the longer effect and highest percentage of individuals with enhanced cortical excitability.

**Conclusion:**

The results suggest that aiTBS protocols at different time intervals result in different motor cortical excitability after-effects.

## Highlights

- Accelerated TBS attracts attention in clinical settings.
- The study examined cortical plasticity induced by 3 sessions of iTBS at different intervals.
- Time interval affects cortical plasticity of accelerated TBS significantly.

## Introduction

Transcranial magnetic stimulation (TMS) is a non-invasive neural regulation technique that can be used to modulate cortical excitability in the brain (Nordmann et al., 2015). Theta-burst stimulation (TBS) is a patterned repetitive paradigm that evokes cortical plasticity in a much shorter time period (Huang et al., 2005). Intermittent TBS (iTBS) can up-regulate cortical excitability and induces long-term potentiation (LTP). Conversely, continuous TBS (cTBS) down-regulates cortical excitability and induces long-term depression (LTD)–like effects (Wischnewski and Schutter, 2015). The after-effect of iTBS is time-varying; reportedly after a single iTBS, the maximum excitatory cortical effect is reached within 10 min, and the excitatory effect gradually returns to the initial state after 30 min of iTBS (Wischnewski and Schutter, 2015; Chung et al., 2016).

Recently, it is proposed that accelerated iTBS (aiTBS), where repeated blocks of iTBS are applied, might bring improved clinically beneficial (Caeyenberghs et al., 2019). This might be due to its stronger induction effects on cortical plasticity. Interestingly, it is proposed that longer iTBS protocol with two blocks of 600 pulses iTBS does not elicit the similar cortical plasticity as single iTBS; for instance, the plasticity reverses (Gamboa et al., 2010). This result might be explained by metaplasticity effect – induction of new synaptic plasticity is dependent on historical synaptic activity (e.g., dosage, time interval of previous protocols) (Muller-Dahlhaus and Ziemann, 2015). However, few studies systematically examined the importance of time interval among different TBS sessions.

Studies have shown that the cortical excitatory effect of iTBS is dose-dependent, three-serially blocks of iTBS applied to the motor cortex; the effect of increasing cortical excitability was significantly higher than that of two blocks of iTBS (Nettekoven et al., 2014, 2015). However, previous studies have focused on exploring the effects of two repeated blocks of iTBS with different intervals or single iTBS (Gamboa et al., 2011; Chung et al., 2018; Tse et al., 2018); repeated triple blocks of iTBS studies are lacking.

Williams et al. (2018) first stated that 10 sessions of iTBS1800 per day are an effective treatment for refractory depression; this aiTBS protocol was then named Stanford Accelerated Intelligent Neuromodulation Therapy (SAINT) (Cole et al., 2019). The present study compared the effects of triple blocks of iTBS600 (total 1,800 pulses per session) at different time intervals (0, 10, and 30 min) on motor cortical excitability in a crossover design. Our main purpose was to identify potentially optimized time intervals for aiTBS applications, in order to maximize the evoked cortical plasticity in terms of motor-evoked potentials (MEPs) amplitude, lasting effect, and minimum individual differences. The secondary purpose was to understand its mechanism by observing the tendency of iTBS1800 in regulating cortical excitability.

## Materials and Methods

### Participants

Sixteen healthy male volunteers, aged from 20 to 30 years (mean and SD = 22.75 ± 2.62 years), were recruited for the present study (Table 1). All participants were right-handed, as verified using the Edinburgh Handedness Inventory (Oldfield, 1971). None of the participants had any history of neurological or psychiatric disorders, serious illnesses, or epilepsy or took potentially hazardous drugs before the application of TMS. Participants provided informed consent prior to the experiment, and the experimental protocol was approved by Huashan Institutional Review Board.

**TABLE 1:** Basic information of subjects.

Subjects	Age (years)	Gender	Dominant hemisphere	Stimulation site
1	21	M	L	LM1
2	22	M	L	LM1
3	28	M	L	LM1
4	24	M	L	LM1
5	21	M	L	LM1
6	22	M	L	LM1
7	22	M	L	LM1
8	22	M	L	LM1
9	22	M	L	LM1
10	23	M	L	LM1
11	20	M	L	LM1
12	30	M	L	LM1
13	22	M	L	LM1
14	21	M	L	LM1
15	22	M	L	LM1
16	21	M	L	LM1
Mean	22.75			
SD	2.62			

### Experiment Design

**FIGURE 1:**
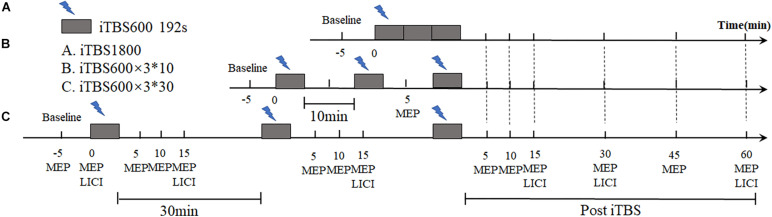
Experimental design. Using a within-subjects cross-over design, each subject took part in three sessions in different day. MEPs and LICI were assessed before and post iTBS intervention. The time required for **(A)** iTBS1800 to received iTBS protocol was 9 min and 52 s. **(B)** iTBS600 × 3^∗^10 was required to receive three blocks of iTBS stimuli, each of which was 10 min apart. the total stimulus time was 29 min and 36 s. **(C)** iTBS600 × 3^∗^30 received three blocks of iTBS stimuli at a 30-min interval, the total stimulus time was 69 min and 36 s.

This study is a single-blind, crossover design. Each subject received iTBS protocols in three different regimens with randomized sequences: continuous 1,800 pulse iTBS stimulation (iTBS1800), three blocks of iTBS with 10-min interval (iTBS600 × 3^∗^10), and three blocks of iTBS with 30-min interval (iTBS600 × 3^∗^30). Each session was 1 week apart to avoid potential lasting effects (Figure 1).

### TMS and EMG Procedure

Subjects were advised to get enough sleep, avoid strenuous exercise, or drink stimulating beverages (e.g., coffee or tea) before taking part in the experiment. Before the first iTBS session, participants were told relevant experimental procedures; they underwent a thorough TMS safety screening procedure, and written informed consent was obtained. The experiment was conducted in a separate, quiet room. Participants were seated in a comfortable position on a recliner with armrests and were instructed to relax. The participants then wore a positioning scalp cap to reduce sliding between the hair and the stimulus coil. During the entire experiment, the environment was quiet, and the participants were not allowed to talk, sleep, or use their mobile phones.

Single TMS to the left primary motor cortex (LM1), which controls the right hand, was performed using an OSF-pTMS magnetic stimulator (O.SELF Company, Wuhan, China) with a figure-of-eight–shaped coil. The stimulating coil was held tangentially to the skull with the coil handle pointing backward and laterally 45° away from the anterior–posterior axis. To assess the motor cortex excitability, a surface electromyography (EMG) was recorded by attaching a pair of Ag-Ag/Cl electrodes to the first dorsal interosseous (FDI) muscle of the participants’ right hand. Participants’ muscle relaxation was observed by visual and EMG monitoring.

Cortical excitability was assessed by measuring the peak-to-peak amplitude of MEPs from the non-dominant FDI at rest. The stimulation intensities (as a percentage of maximal stimulator output) of TMS were increased to evoke MEP_rightFDI_ wave 1-mV peak-to-peak (SI_1mV_); participants received 10 consecutive single pulses to the target point of the left M1 at an interval of 5 s. Furthermore, to measure MEPs, the intensity remained constant at baseline intensity throughout each experimental session. Resting motion threshold (RMT) was defined as the minimal stimulation intensity that could induce at least 5 of 10 consecutive trials in the FDI muscle with MEPs peak-to-peak wave amplitude greater than 50 μV. Participants’ RMT and MEPs were remeasured before each session. To ensure the intraindividual reliability of cortical excitability, we recorded 2 sessions of MEP measurements of each participant, with a 5-min interval at baseline. once the average amplitude difference between the two measurements was less than 20%, the experiment could begin. If not, the baseline MEPs was measured until, for two consecutive times, the average amplitude difference of MEPs was less than 20%.

For three different iTBS protocols, MEPs were recorded by 10 single pulses with 1-mV stimulus intensities (SI_1mV_). And it should be measured twice at baseline and at time points 5, 10, 15, 30, 45, and 60 min post-1,800 pulses. A total of eight measures of cortical excitability were required for iTBS1800. Furthermore, two sessions had break between interventions; MEPs were also measured at intervention intervals extra. The MEPs of iTBS600 × 3^∗^10 secession were recorded at 5 min when there was a break between interventions. A total of 10 measurements of cortical excitability were required for this protocol, and iTBS600 × 3^∗^30 session MEPs were recorded at 5, 10, and 15 min during intervention interval times. A total of 14 measurements of cortical excitability were required for this protocol.

Long interval intracortical inhibition (LICI) was assessed using a paired-pulse TMS protocol. In a very short period (100 or 150 ms), two consecutive stimuli were performed in the same hemisphere. The intensity of both conditioning stimulus (CS) and test stimulus (TS) was above threshold. The conditioning pulse inhibited the original MEP. We tested LICI with a CS intensity set at 120% of RMT; the TS intensity was the same as the measured cortical excitability that could evoke an MEP_rightFDI_ of l-mV amplitude. The interstimulus interval was 150 ms. Ten trials with single pulses (unconditioned) and 10 trials with paired pulses (conditioned) were recorded at an alternating order with an interval of 5 s. The intensity of both conditional and stimulus pulses was above the threshold. LICI was measured at 15 and 60 min after each protocol of 1,800 pulses iTBS. For iTBS600 × 3^∗^30, LICI was also measured at time point of 15 min between the interval of two blocks of iTBS.

### Intermittent Theta-Burst Stimulation

The iTBS pattern consisted of bursts containing three pulses at 50 Hz repeated at 5 Hz; 2-s train of TBS was repeated every 10 s for a total of 192 s (600 pulses) (Huang et al., 2005). The stimulation intensity of the experiment was set at 70% of RMT.

### Statistics Analysis

SPSS version 22 (Statistical Package for the Social Sciences; IBM, Armonk, NY, United States) software was used for the statistical analysis of the data. To calculate plasticity, the MEPs were normalized to baseline MEP amplitude for each participant. LICI was expressed as the ratio of conditioned MEPs to unconditioned MEPs. All data (participant’s age, RMT, SI_1mV_, MEP amplitude, MEP latency, and LICI) were expressed as mean ± SD. One-way analysis of variance (ANOVA) was used to compare RMT, S1mV, MEP amplitude, and LICI at baseline between different sessions. Each time point after each iTBS protocol was compared with the baseline used paired *t* test to analyze the after-effects of each iTBS conditions. To examine the effect of different iTBS intervals on cortical excitability and inhibition, repeated-measures ANOVAs (reANOVAs) were used to test the main effects of TREATMENT (iTBS1800, iTBS600 × 3^∗^10, iTBS600 × 3^∗^30) and TIME (baseline, 5, 10, 15, 30, 45, 60) on MEP amplitude and LICI. The sphericity was verified used the Mauchly test; when not met, Greenhouse–Geisser was used to correct for this. Pearson correlations were used to assess the relationship between the LTP-like plasticity induced by different TMS conditions, whereas the correlation was determined between LICI of baseline and mean MEP amplitude at 10, 30, and 60 min after iTBS. The correlation between the change of LICI and MEP amplitude at 15 and 60 min compared to baseline was also calculated by Pearson correlation test. Statistically significant values are defined as *P* < 0.05.

## Results

All participants completed three sessions in the study, and no side effects were reported by the subjects during or after the experimental sessions. No significant differences were found when the ANOVAs were performed to test RMT (*F*_2,15_ = 0.01, *p* = 0.990), MEP amplitude (*F*_2,15_ = 1.27, *p* = 0.290), SI_1mv_ (*F*_2,15_ = 0.03, *p* = 0.975), or LICI (*F*_2,15_ = 0.66, *p* = 0.521) among the three visits (Table 2).

**TABLE 2:** Subjects’ baseline RMT, MEP amplitude, SI_1mv_, and LICI when started three different iTBS conditions.

	iTBS1800 (*N* = 16)	iTBS600 × 3^∗^10 (*N* = 16)	iTBS600 × 3^∗^30 (*N* = 16)	*F*	*P*
RMT (%MSO)	39.69 ± 15.99	40.31 ± 14.70	40.38 ± 14.45	0.010	0.990
MEP (mV)	1.03 ± 0.23	1.22 ± 0.43	1.14 ± 0.35	1.272	0.290
SI_1mv_ (%MSO)	50.8 ± 20.05	51.88 ± 19.82	52.25 ± 17.19	0.025	0.975
LICI	0.37 ± 0.46	0.22 ± 0.35	0.24 ± 0.41	0.662	0.521

### Temporal Changes of Cortical Excitability in Different iTBS Protocol

**FIGURE 2:**
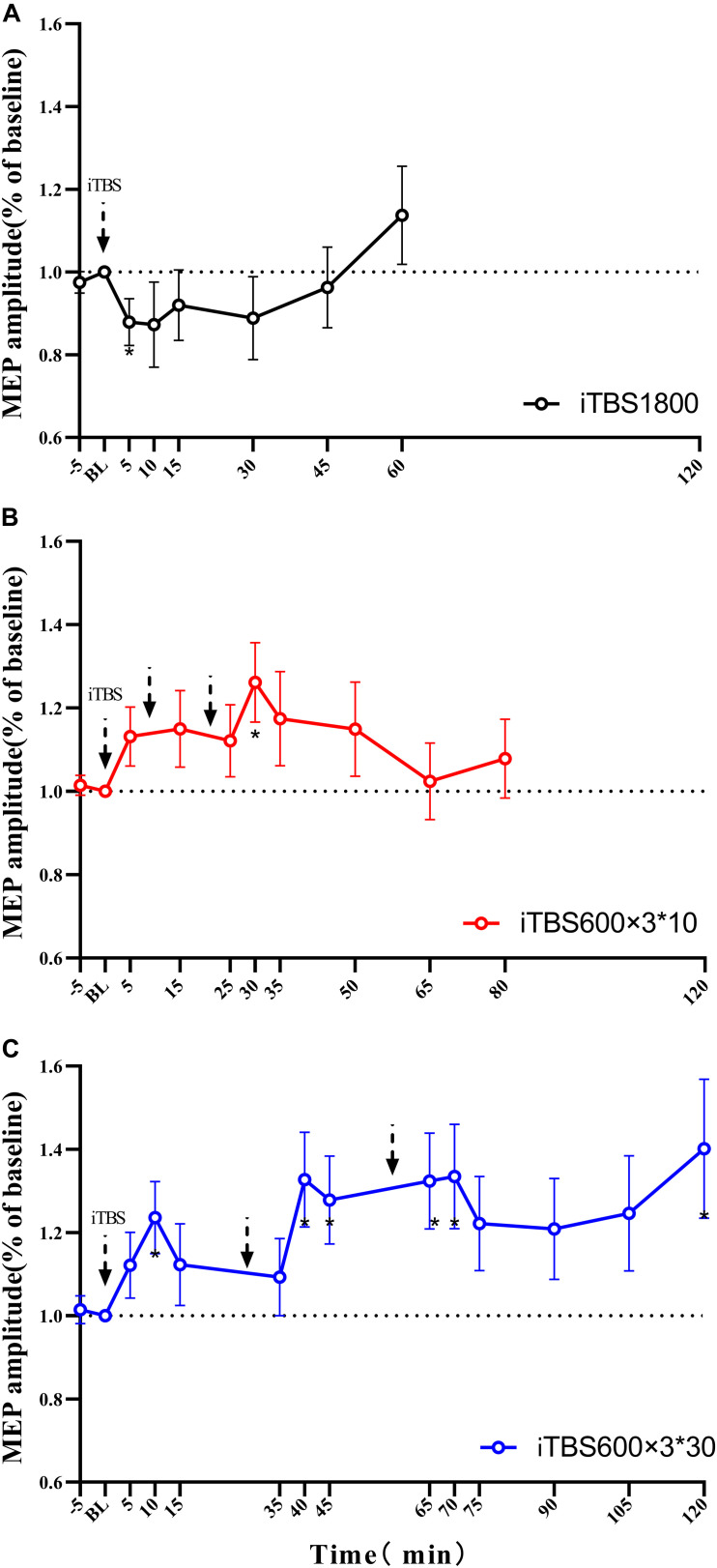
MEPs amplitude at stimulation interval after each block of iTBS and up to 60 min post 1800 pulses of iTBS in difference iTBS conditions. For each cluster of **(A)** iTBS1800 **(B)** iTBS600 × 3^∗^10 **(C)** iTBS600 × 3^∗^30. Asterisks indicate statistical significance between the MEPs amplitude at that time point and the baseline MEPs amplitude (*P<0.05).

The duration of the after-effects of each individual iTBS protocol was examined by pairing *t* test to baseline. Instead of inducing facilitation, the iTBS1800 suppressed MEP amplitude, which was significant at the 5-min time point (*p* = 0.049). However, the MEP amplitude gradually recovered to its baseline, and facilitation occurred at 50 min post–1,800 pulses (Figure 2A).

For the iTBS600 × 3^∗^10 group, the facilitation effects on MEPs amplitude were significant at time point 30 min (*p* = 0.015) from the baseline. The MEPs amplitude at time 5 min post–1,800 pulses was slightly larger than 5 min after 600 and 1,200 pulses stimulus, but statistically not significant (*p* = 0.914, *p* = 0.728, respectively) (Figure 2B).

For the iTBS600 × 3^∗^30 group, the facilitation effect was still evident after 120 min. In this iTBS paradigm, significant differences were seen if compared to the baseline at the time point 10 min (*p* = 0.016) after 600 pulses; 10 min (*p* = 0.012) and 15 min (*p* = 0.019) after 1,200 pulses; and 5 min (*p* = 0.013), 10 min (*p* = 0.018), and 60 min (*p* = 0.030) after 1,800 pulses (Figure 2C).

### After-Effect of 1,800-Pulse Dosage

Two-way reANOVAs were employed to compare the differences across the three groups: MEP responses were aligned to the completion of 1,800 pulses (Table 3). The results revealed a significant main effect of the factors TREATMENT (*F*_2,30_ = 3.734, *p* = 0.036), TIME (*F*_6,90_ = 2.886, *p* = 0.013), and the interaction TREATMENT × TIME (*F*_12,180_ = 2.004, *p* = 0.026). For the interaction, we analyzed the simple effect of TREATMENT and TIME separately by one-way reANOVAs. For the factors of TREATMENT, there were significant simple effect at 5 min (*F*_2,30_ = 7.423, *p* = 0.002) and 10 min (*F*_2,30_ = 5.715, *p* = 0.008) post–1,800 pulse iTBS; for the factor of TIME, significance was found only in iTBS600 × 3^∗^30 group (*F*_6,90_ = 2.609, *p* = 0.022).

**TABLE 3:** Normalized MEP amplitude for baseline and post stimulation measurement.

TBS intervention protocol	MEP amplitude (MV)						
	Time after stimulation (min)						
	Baseline	T5	T10	T15	T30	T45	T60
iTBS1800 (*N* = 16)	1.00 ± 0.00	0.88 ± 0.23	0.87 ± 0.41	0.92 ± 0.34	0.89 ± 0.40	0.96 ± 0.39	1.14 ± 0.48
iTBS600 × 3^∗^10 (*N* = 16)	1.00 ± 0.00	1.12 ± 0.35	1.26 ± 0.38	1.17 ± 0.45	1.15 ± 0.45	1.02 ± 0.37	1.08 ± 0.38
iTBS600 × 3^∗^30 (*N* = 16)	1.00 ± 0.00	1.32 ± 0.46	1.33 ± 0.50	1.22 ± 0.45	1.21 ± 0.48	1.25 ± 0.55	1.40 ± 0.67

**FIGURE 3:**
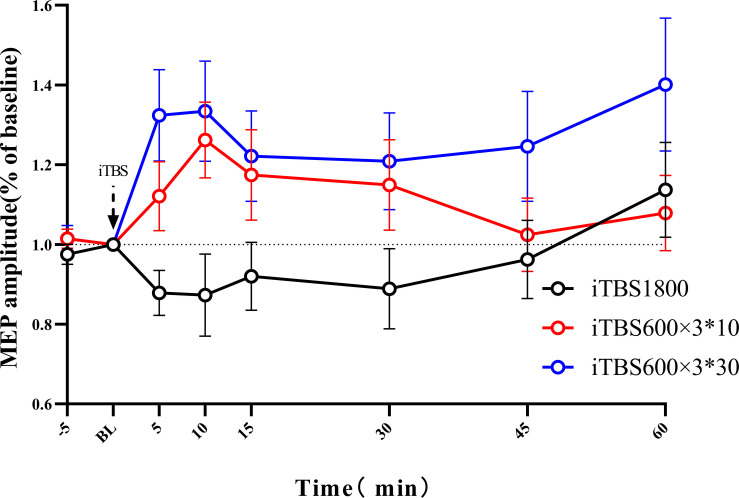
The after-effect of 1800 pulses iTBS on MEP amplitude at times 5, 10, 15, 30, 45, and 60 min following three different iTBS condition. MEPs amplitude were normalized to baseline.

According to the ANOVAs results, there is a significant difference in TREATMENT at the time points 5 min (*p* = 0.004) and 10 min (*p* = 0.009). *Post hoc* with Bonferroni correction revealed significant differences between iTBS1800 and iTBS600 × 3^∗^30 at 5 min (*p* = 0.003) and 10 min (*p* = 0.013) and significant differences between iTBS1800 and iTBS600 × 3^∗^10 at 10 min (*p* = 0.045) (Figure 3).

### Individual Exhibits Distinct Plasticity Responses to Different Protocols

**FIGURE 4:**
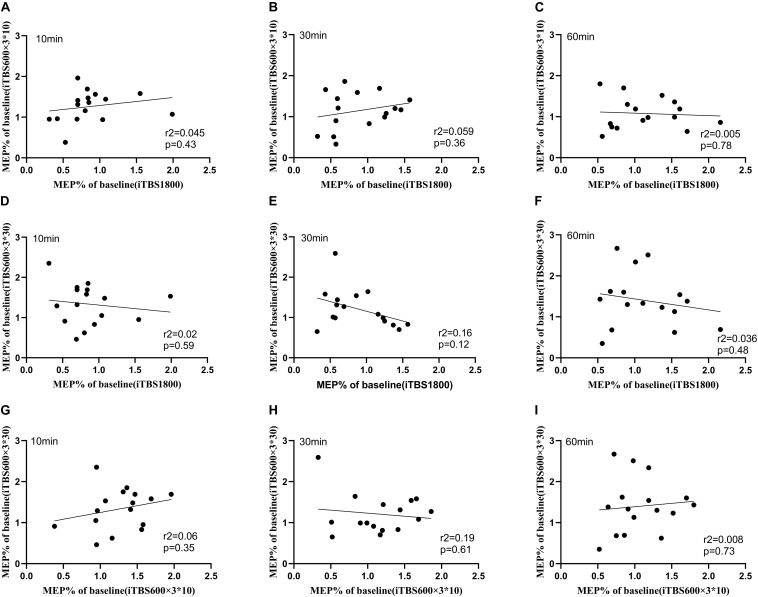
Relationship between iTBS1800, iTBS600 × 3^∗^10, and iTBS600 × 3^∗^30 at time points 10, 30, and 60 min. Correlation between the normalized grand average MEPs amplitude following different iTBS conditions **(A)** MEPs amplitude correlation of iTBS1800 and iTBS600 × 3^∗^10 at 10 min **(B)** MEPs amplitude correlation of iTBS1800 and iTBS600 × 3^∗^10 at 30 min **(C)** MEPs amplitude correlation of iTBS1800 and iTBS600 × 3^∗^10 at 60 min **(D)** MEPs amplitude correlation of iTBS1800 and iTBS600 × 3^∗^30 at 10 min **(E)** MEPs amplitude correlation of iTBS1800 and iTBS600 × 3^∗^30 at 30 min **(F)** MEPs amplitude correlation of iTBS1800 and iTBS600 × 3^∗^30 at 60 min **(G)** MEPs amplitude correlation of iTBS600 × 3^∗^10 and iTBS600 × 3^∗^30 at 10 min **(H)** MEPs amplitude correlation of iTBS600 × 3^∗^10 and iTBS600 × 3^∗^30 at 30 min **(I)** MEPs amplitude correlation of iTBS600 × 3^∗^10 and iTBS600 × 3^∗^30 at 60 min.

We checked if one individual exhibited similar levels of plasticity to the three types of TBS protocols. Hence, the Pearson correlation test was used to assess the correlation among the three sessions. No correlation for cortical plasticity was identified between the three sessions (Figure 4).

### Effect of iTBS on Intracortical Inhibition

We used reANOVAs to see if different iTBS conditions affected the inhibitory intracortical interneuronal circuitry. The reANOVAs for LICI, at each time point in the experiment, showed no effects correlated to the TREATMENT (*F*_2,30_ = 1.530, *p* = 0.233), TIME (*F*_2,30_ = 2.135, *p* = 0.136), or TREATMENT × TIME (*F*_4,60_ = 1.157, *p* = 0.332) interaction.

### Interindividual and Intraindividual Effects

**FIGURE 5:**
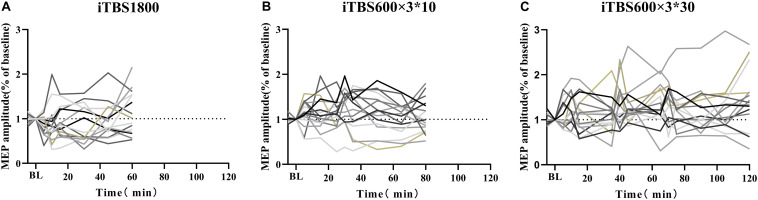
Individual response to iTBS after 1800 pulses stimulation in three different intervals. **(A)** iTBS1800 **(B)** iTBS600 × 3^∗^10 **(C)** iTBS600 × 3^∗^30.

**FIGURE 6:**
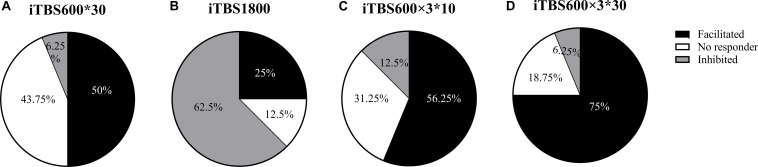
The percentage of subjects that responded to different iTBS conditions. **(A)** one blocks of iTBS **(B)** iTBS1800 **(C)** iTBS600 × 3^∗^10 **(D)** iTBS600 × 3^∗^30.

The individual responses to the three different protocols were plotted (Figure 5). In addition, when the multiple time point average value was >1.2, it was considered as facilitation responder; when multiple time point average value was less than 0.8, it was considered as inhibitory responders; those who compared the change in MEP to baseline between 1.2 and 0.8 were considered no responders. We took the response of the subjects to the first 30 min in iTBS600 × 3^∗^30 group as a classic iTBS response, and the percentage of subjects showing a facilitation was 50% (Figure 6A). In the iTBS1800, 25% of subjects showed facilitation, whereas 62.5% showed inhibition (Figure 6B). In the iTBS600 × 3^∗^10, 56.25% of subjects showed facilitation, and 12.3% were inhibited, respectively (Figure 6C). The facilitation effect of iTBS600 × 3^∗^30 was more obvious than the other two iTBS conditions; 75% of subjects showed facilitation, and 6.25% were inhibited (Figure 6D).

## Discussion

This study is an exploratory study to identify a clinically effective protocol for aiTBS and understand the effect and mechanism of iTBS1800 on cortical excitability. We reported that the dose-dependent accumulative effect for cortical plasticity is affected by the interval from each iTBS session. Considering the potential side effects induced from total dosage, the 30-min interval three blocks of iTB600 protocol exhibited the longest potentiation effects on the motor cortex. There was no significant inhibitory effect on cortical excitability in iTBS1800, but 50 min after 1,800 pulses of iTBS, the facilitation effect gradually appears.

This is the first study where continuous 1,800 pulses of iTBS to M1 are performed. Within 50 min of the stimulus, the after-effects of iTBS1800 were the same as previous studies that performed iTBS1200 (Gamboa et al., 2010) and cTBS (Guerra et al., 2019), which inhibits cortical excitability. Because we did not compare the iTBS1200 group and classical iTBS group, we could not compare the inhibitory effect of the three protocols. However, contrary to our findings, another study that prolonged the theta burst stimulation to 1,200 pulses (Hsu et al., 2011) showed that iTBS1200 enhanced facilitation and time course of corticospinal tract. The different results may be due to the different stimulus dose and because we conducted iTBS1800 immediately after the RMT was measured, whereas in the experiment of Hsu et al. (2011) there was a 5-min rest after RMT was measured. The excitatory effect begins after 50 min of stimulation. Because our experiment was not observed for a longer time, the reasons behind the change in amplitude are unclear. This is consistent with other studies where previously saturated LTP was significantly enhanced by several multiblocks of iTBS to the cerebral cortex if the interval was more than 50 min (Kramár et al., 2012; Thomson et al., 2019). Our research could explain the mechanism by which the SAINT protocol [10 sessions of iTBS1800 with interval of 50 min to the left dorsolateral prefrontal cortex (DLPFC)] effectively treats refractory depression. Although the cortical areas we stimulated were different from those used to treat depression, the effect of iTBS1800 on cortical excitability is consistent.

For classical iTBS, the MEP amplitude is maximized at 10–15 min post-iTBS, and excitation gradually decreased (Wischnewski and Schutter, 2015). In our study, we have chosen the time interval of 10 and 30 min, compared to iTBS1800 and classical iTBS. We found that repeated blocks of iTBS did have a cumulative effect when the appropriate intervals were selected. Previous studies demonstrated that cortical plasticity evoked by 1,200 pulses of iTBS did not mimic a single session of iTBS (600 pulses), depending on the time interval between the 2 iTBS sessions. No and 5-min intervals led to depression (Gamboa et al., 2011; Tse et al., 2018), whereas 15-min interval led to a potentiation similar to as a single session (Nettekoven et al., 2014). In addition, 15-min interval of three sessions of iTBS induced enhanced cortical plasticity than a single session of iTBS (Nettekoven et al., 2014), which is consistent in our results. All these results suggested that an aiTBS sessions should have an interval larger than 15 min to prevent depression-like plasticity responses.

The superposition and reversal of the after-effect of repeated blocks of iTBS may be explained by the following hypothesis. The effects of TBS are dependent on *N*-methyl-D-aspartate receptors (Huang et al., 2007), which allows calcium ions to enter the postsynaptic membrane and trigger LTP or LTD (Citri and Malenka, 2008). This influx triggers the generation of LTP, and the increased concentration of calcium ions in the postsynaptic membrane is one of the necessary conditions for the formation of LTP (Cho et al., 2001; Abraham, 2008). Conversely, both LTP and LTD are triggered by the same second messenger Ca^2+^. There is a calcium level called “no man’s land” between the LTP and LTD Ca^2+^ zones that results in no plasticity. Whether LTP or LTD occurs depends on the Ca^2+^ levels after TBS protocol (Lisman, 2001). Conversely, a theory of synaptic metaplasticity was proposed by Fung and Robinson (2014). They simulated the oscillations of intracellular calcium, calcium conductance, and plasticity signals. The results showed that the relationship between the after-effect with stimulus dose and time of iTBS presented waveforms; inhibition and facilitation alternate with the change of stimulus dose and intervention time.

Intracortical inhibition (ICI) and intracortical facilitation (ICF) measurements are commonly used to explore intracortical inhibition and facilitatory circuits. Short interval intracortical inhibition (SICI) and LICI were mediated via postsynaptic γ-aminobutyric acid (GABA) A and B receptors, respectively; LICI leads to a long-lasting inhibitory postsynaptic potential and described as late cortical disinhibition (Werhahn et al., 1999; Cash et al., 2010). Previous studies suggested that SICI significantly increased or reduced following iTBS and cTBS, whereas ICF did not change after both TBS protocols (Huang et al., 2005; Murakami et al., 2008). According to a meta-analysis, SICI changes only occur in a very short time point (<5 min) after cTBS; no significant differences were found in SICI at any time point with iTBS (Chung et al., 2016). There were no changes in SICI following classical iTBS and break repeat blocks of iTBS (Tse et al., 2018). In our study, no changes in LICI were found after iTBS intervention or at different intervals of iTBS conditions.

Previous studies also reported high intersubject variability in cortical plasticity studies. The subjects were given the same iTBS protocols; 43% of the subjects were responders that increase the MEPs amplitude, whereas 57% were non-responders (Lopez-Alonso et al., 2014). Furthermore, the same subject exhibited different after-effects to iTBS on their different visits (Schilberg et al., 2017). One study confirmed a relationship between the dose of stimulation and individual responses to TMS (Fitzgerald et al., 2020). The response to repeated iTBS varies depending on the individual (Tse et al., 2018). In our current study, only 25% of subjects showed facilitation in TBS1800, but an increase to 56.25% of subjects showed facilitation in iTBS600 × 3^∗^10. The facilitation effect of iTBS600 × 3^∗^30 was more obvious than the other two iTBS conditions where 75% of subjects showed facilitation. The results suggest that an individual’s response to TBS could also be modulated by prior TBS exposures. Furthermore, there is no correlation between the after-effect of each iTBS condition at any time point, indicating that the mechanism of different schemes may be different, and the response of different individuals to different iTBS schemes may be different.

There are several limitations in our study. First, we measured cortical excitability and cortical inhibition only within 60 min of iTBS protocols. It is unclear what happens after 60 min; for iTBS1800, the cortical excitability after 60 min shifted toward greater facilitation or a return to the original state. Second, we tested only the direct effect of aiTBS on M1 and recruited only young males in order to reduce the interindividual variability. Therefore, more studies are needed to confirm the effect of aiTBS on different brain regions (e.g., DLPFC, cerebellum, and Broca) and other healthy subjects, as well as patients.

In conclusion, the varied time intervals of iTBS sessions contributed to distinct cortical plasticity responses in aiTBS protocols. Our data indicate that three blocks of iTBS with a 30-min interval induce prominent cortical plasticity. We suggest that it might be necessary to test patients’ response to iTBS protocols in order to improve clinical efficacy.

## Data Availability Statement

The original contributions presented in the study are included in the article/supplementary material, further inquiries can be directed to the corresponding authors.

## Ethics Statement

The studies involving human participants were reviewed and approved by the Huashan Hospital Affiliated to Fudan University Institutional Review Board (HIRB). The patients/participants provided their written informed consent to participate in this study.

## Author Contributions

FY: conceptualization, methodology, validation, formal analysis, investigation, data curation, writing – original draft, and visualization. XT: software and investigation. RH: resources, supervision, and funding acquisition. SL, WW, and ST: investigation. YW: project administration. T-FY: conceptualization, methodology, writing – review and editing, and supervision. YZ: resources, supervision, funding acquisition, writing – review and editing, project administration, and supervision. All authors contributed to the article and approved the submitted version.

## Conflict of Interest

The authors declare that the research was conducted in the absence of any commercial or financial relationships that could be construed as a potential conflict of interest.
