# Corrigendum: The After-Effect of Accelerated Intermittent Theta Burst Stimulation at Different Session Intervals

**DOI:** 10.3389/fnins.2021.687972

**Published:** 2021-05-05

**Authors:** Fengyun Yu, Xinwei Tang, Ruiping Hu, Sijie Liang, Weining Wang, Shan Tian, Yi Wu, Ti-Fei Yuan, Yulian Zhu

**Affiliations:** ^1^Department of Rehabilitation Medicine, Huashan Hospital, Fudan University, Shanghai, China; ^2^School of Kinesiology, Shanghai University of Sport, Shanghai, China; ^3^Shanghai Key Laboratory of Psychotic Disorders, Shanghai Mental Health Center, Shanghai Jiao Tong University School of Medicine, Shanghai, China; ^4^Co-innovation Center of Neuroregeneration, Nantong University, Nantong, China

**Keywords:** theta burst stimulation, accelerated, motor cortex, cortical plasticity, stimulation interval

In the original article, there was a mistake in the legend for **Figure 5(C)** and **Figure 6(D)** as published. It should be “iTBS600×3*30,” but it was written incorrectly to “iTBS×3*10.” The correct legend appears below.

**Figure 5**. Individual response to iTBS after 1800 pulses stimulation in three different intervals. **(A)** iTBS1800 **(B)** iTBS600 × 3*10 **(C)** iTBS600 × 3*30.

**Figure 6**. The percentage of subjects that responded to different iTBS conditions. **(A)** one blocks of iTBS **(B)** iTBS1800 **(C)** iTBS600 × 3*10 **(D)** iTBS600 × 3*30.

In the original article, there was a mistake in Table 2 as published. The corrected Table 2 appears below. The SI1mv data for iTBS600×3*10 was incorrectly filled in as 198.2.

**Table 2:** Subjects' baseline RMT, MEP amplitude, SI_1mv_, and LICI when started three different iTBS conditions.

	**iTBS1800 (*N* = 16)**	iTBS600 × 3*10 (*N* = 16)	iTBS600 × 3*30 (*N* = 16)	***F***	***P***
RMT (%MSO)	39.69 ± 15.99	40.31 ± 14.70	40.38 ± 14.45	0.010	0.990
MEP (mV)	1.03 ± 0.23	1.22 ± 0.43	1.14 ± 0.35	1.272	0.290
SI_1mv_ (%MSO)	50.8 ± 20.05	51.88 ± 19.82	52.25 ± 17.19	0.025	0.975
LICI	0.37 ± 0.46	0.22 ± 0.35	0.24 ± 0.41	0.662	0.521

The authors apologize for this error and state that this does not change the scientific conclusions of the article in any way. The original article has been updated.

